# Nonsex Genes in the Mating Type Locus of *Candida albicans* Play Roles in a/α Biofilm Formation, Including Impermeability and Fluconazole Resistance

**DOI:** 10.1371/journal.ppat.1002476

**Published:** 2012-01-12

**Authors:** Thyagarajan Srikantha, Karla J. Daniels, Claude Pujol, Nidhi Sahni, Song Yi, David R. Soll

**Affiliations:** The Developmental Studies Hybridoma Bank, Department of Biology, University of Iowa, Iowa City, Iowa, United States of America; Carnegie Mellon University, United States of America

## Abstract

The mating type locus (*MTL*) of *Candida albicans* contains the mating type genes and has, therefore, been assumed to play an exclusive role in the mating process. In mating-incompetent **a**/α cells, two of the mating type genes, *MTL* **a**1 and *MTL*α2, encode components of the **a**1-α2 corepressor that suppresses mating and switching. But the *MTL* locus of *C. albicans* also contains three apparently unrelated “nonsex” genes (NSGs), *PIK*, *PAP* and *OBP*, the first two essential for growth. Since it had been previously demonstrated that deleting either the **a**/α copy of the entire *MTL* locus, or either *MTL*a1 or *MTL*α2, affected virulence, we hypothesized that the NSGs in the *MTL* locus may also play a role in pathogenesis. Here by mutational analysis, it is demonstrated that both the mating type and nonsex genes in the *MTL* locus play roles in **a**/α biofilm formation, and that *OBP* is essential for impermeability and fluconazole resistance.

## Author Summary

Most natural strains of the yeast pathogen *Candida albicans* are diploid and heterozygous (**a**/α) at the mating type locus (*MTL*). The *MTL* locus contains mating type genes and has been assumed to play roles exclusively in the mating process of **a/a** and α/α cells. In *C. albicans*, however, the *MTL* locus also contains three nonsex genes (NSGs), the essential phosphatidyl inositol kinase gene, *PIK*, the essential poly(A) polymerase gene, *PAP*, and the nonessential oxysterol binding protein gene, *OBP*. We demonstrate for the first time that both the mating type genes *MTL* **a**1 and *MTL*α2, and the three NSGs play non-mating roles in **a**/α biofilm formation and virulence. In addition, we show that the NSG *OBP* is necessary for impermeability and fluconazole resistance of **a**/α biofilms. These results demonstrate that nonsex genes as well as two mating type genes embedded in the mating type locus, play related roles in pathogenic processes unrelated to mating in **a**/α cells.

## Introduction

The **a** and α copies of the mating type locus (*MTL*) of *Candida albicans* contain several genes that play highly specialized roles in the mating process, *MAT* **a**1 and *MAT* **a**2 in the **a** copy, and *MAT*α1 and *MAT*α2 in the α copy [1]. This locus also contains three genes apparently unrelated to mating, the nonsex genes (NSGs) *PIK, PAP* and *OBP* [1]–[3]. Other members of the *Candida* clade of the hemiascomycetes also contain these NSGs in their *MTL*s [2], but members of the *Saccharomyces* clade, including *Saccaromyces cerevisiae*, do not [4]. Interestingly, the mating type loci of several other fungi contain NSGs [5]–[8]. The mating type locus of *Cryptococcus neoformans*, for instance, contains approximately 20 genes, including mating type genes, mating-related genes and NSGs, both essential and nonessential [5], [6]. However, there is no evidence in these other fungi that the NSGs serve related functions or play roles in mating. In *C. albicans*, the NSG *PIK* is an essential gene that encodes a phophatidylinositol kinase involved in signal transduction [9]–[12]. The NSG *PAP* is an essential gene encoding the poly(A) polymerase, which polymerizes adenosine (A) at the 3′ ends of mRNAs [13]–[16]. The NSG *OBP* is the only nonessential gene, encoding an oxysterol binding protein involved in sterol sensing in the cytoplasm [17]–[21]. In *C. albicans*, *PIK*, *PAP*, and *OBP* are not only located in the *MTL* locus [3], but the **a** and α alleles of each have diverged at rates far higher than those of other genes bordering the *MTL* locus, a characteristic of NSGs in the mating type loci of other fungi [6]–[8].

Until recently it had been assumed that in mating-incompetent **a**/α cells, the mating-related genes in the *MTL* locus play exclusive roles in repressing white-opaque switching and mating [22]. No attention had been given to the possibility that the three NSGs played a role in mating or in a common function unrelated to mating. Their known functions predicted no apparent direct role in the mating process. Two of the three genes, *PIK* and *PAP*, were demonstrated to be essential for growth in *S. cerevisiae* [9], [13] and, therefore, presumably essential in *MTL* heterozygous and homozygous configurations of *C. albicans*. The mating-type genes in *MTL*, on the other hand, were all nonessential for growth, but *MTL* **a**1 and *MTL*α2 did function to suppress mating in **a**/α cells [1], [23]. In *S. cerevisiae*, there were indications that the orthologs of *C. albicans MTL* **a**1 and *MTL*α2 also played non-mating roles in wall maintenance during the stress response [24] and that *MAT*α2 played a role in invasive growth [25]. In *C. albicans* there were also indications that genes in the *MTL* locus played non-mating roles. The configuration of the *MTL* locus was implicated in virulence in a mouse model for systemic infection. First, it was observed that natural **a**/α strains were on average more virulent than natural **a/a** or α/α strains [26]. Second, it was observed that in every case in which an **a/a** or α/α derivative spontaneously emerged from a natural **a**/α strain during *in vitro* culturing, the parental **a**/α strain was more virulent than the *MTL*-homozygous offspring [26]. Finally, it was observed that deleting either *MTL* ***a*** *1* or *MTLα2* in unrelated **a**/α strains resulted in decreased virulence [26]. These results indicated for the first time that the mating type locus and mating type genes might play nonsex roles in the virulence of mating-incompetent **a**/α cells. Given that the three NSGs were also located in the *MTL* locus, we entertained the possibility that they may also contribute to the increased virulence and pathogenesis of **a**/α strains.

Here we have investigated this hypothesis by analyzing the effects of independently deleting, in two unrelated natural **a**/α strains, the **a** or α copy of the *MTL* locus, *MTL* **a**1 or *MTL*α2, all three **a** alleles of the NSG simultaneously, all three α alleles of NSGs simultaneously, each **a** allele of the three NSGs individually, each α allele of the three NSGs individually and both *OBP* **a** and *OBP*α simultaneously. The results reveal that the sex genes *MTL* **a**1 and *MTL*α2, and the NSGs, located in the mating type locus, play roles in biofilm formation and in the virulence of mating-incompetent **a**/α cell. In the case of the only nonessential NSG, *OBP*, analysis of the homozygous deletion derivative revealed that it played no discernible role in the pheromone response or fusion in the mating process, but it did play a role in the formation of a complete **a/**α biofilm that was both impermeable and drug-resistant, two of the most important traits of an **a**/α biofilm. Together, these results suggest that in mating-incompetent **a**/α cells all of the tested genes in the *MTL* locus play roles in common pathogenic processes that are unrelated to the mating process.

## Results

### Nonsex genes in the *MTL* locus

**Figure 1:**
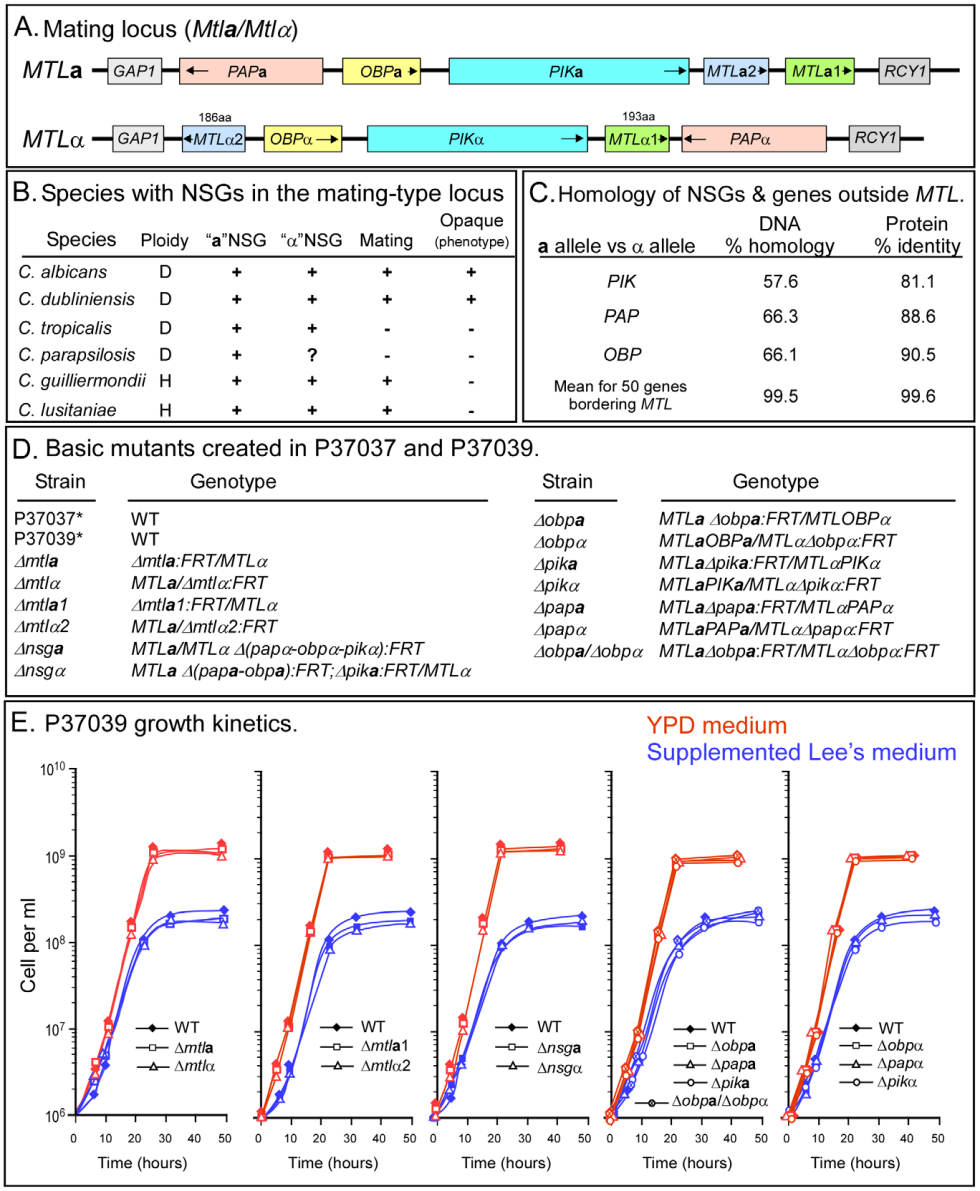
The *MTL* locus of *C. albicans* contains three nonsex genes (NSGs), the a and α alleles of which have diverged. A. The locus contains three NSGs, poly(A)^+^ polymerase (*PAP*), phosphatidyl inositol kinase (*PIK*) and the oxysterol binding protein (*OBP*). The arrow heads in the genes indicate direction of transcription. B. Species in the *Candida* clade of the hemiascomycetes with **a** and α NSGs. C. Comparison of the DNA homology and protein identity of the **a** and α alleles of the NSGs and 50 genes bordering both sides of the *MTL* locus on chromosome 5. D. Genotypes of the parent strains and basic mutants used in this study. E. Growth kinetics in supplemented Lee's medium and YPD medium revealed that none of the mutants used in this study had a growth defect. *These strains were previously described [28]; the remaining mutants were generated for this study.

An **a**/α strain of *C. albicans* harbors an **a** copy of the mating type locus, *MTL*, on one homolog of chromosome 5 and an α copy on the other [1]. The **a** copy of the locus contains the mating genes *MTL* ***a*** *1* and *MTL* ***a*** *2*, as well as the non-sex genes (NSG**a**s) *PIK* ***a***, *PAP* ***a***, and *OBP* ***a***, and the α locus contains *MTLα1* and *MTLα2*, as well as the non-sex genes (NSGαs) *PIKα*, *PAPα* and *OBPα* (Figure 1A) [1], [2]. Three of four diploid species of the *Candida* clade (*C. albicans, Candida dubliniensis, Candida tropicalis*) similarly possess the three NSG**a**s in the *MTL* **a** locus and the three NSGαs in the *MTL*α locus [2], [3] (Figure 1B). In *C. albicans* mating type and non-mating type genes differ both in the direction of transcription and in their relative position in the opposing **a** and α copies of the *MTL* locus (Figure 1A). A fourth diploid species, *Candida parapsilosis,* contains the NSG**a**s in *MTL* **a**, but the composition of *MTL*α is unknown [27] (Figure 1A). *Candida orthopsilosis*, a close relative of *C. parapsilosis*, has both **a** and α loci with NSG**a**s and NSGαs [27]. Two additional *Candida* species (*Candida guilliermondii, Candida lusitaniae*), for which only haploid strains have been identified, contain either the NSG**a**s in an *MTL* **a** locus or the NSGαs in an *MTL*α locus [3] (Figure 1B).

The DNA homologies of the open reading frames of the **a** and α copies of *PIK*, *PAP*, and *OBP* in *C. albicans* are 58, 66 and 66%, respectively, which is in marked contrast to the mean DNA homology of 99.5% computed for the open reading frames of the alleles of 50 genes neighboring the *MTL* locus (Figure 1 C). The identity of the deduced **a** and α proteins encoded by *PIK*, *PAP*, and *OBP* are 81, 89 and 91%, respectively, which again is in marked contrast to the mean identity of 99.6% computed for the deduced proteins encoded by **a** and α proteins of the same 50 genes neighboring the *MTL* locus (Figure 1C).

The homology between the allelic promoter regions of nine genes located upstream of *MTL* and nine genes located downstream were compared with the putative promoter regions of the NSGs. These 18 genes were chosen from the same 50 genes neighboring the *MTL* locus, because they exhibited the most polymorphic open reading frames of the set. The average DNA homology of the promoter regions (700±28 bp) of the alleles of the 18 genes was 96.3%±3.1%. The promoter regions of alleles of all 18 genes revealed unambiguous alignment. However, comparison of the 1 kb or smaller upstream intergenic region of the **a** and α alleles of the NSGs revealed that sequence divergence was too high to support bona fide sequence alignments, and hence, did not allow a measure of homology. These differences indicate that the upstream regulatory regions as well as the open reading frames of the **a** and α copies of the three NSGs have diverged at far higher rates than genes immediately outside the *MTL* locus on chromosome 5.

### Growth rates of all mutants are normal

To test whether *MTL* **a**1, *MTL*α2 and the **a** and α alleles of the NSGs played roles in biofilm formation and virulence, we generated the following deletion mutants in each of two unrelated natural **a**/α strains, P37039 and P37037 [28]: the entire *MTL* **a** locus (Δ*mtl* ***a***); the entire *MTLα* locus (Δ*mtlα*) [26]; *MTL* **a** *1* (Δ*mtl* **a** *1*) [26]; *MTLα2* (Δ*mtlα2*) [26]; all three of the NSG**a** alleles simultaneously, Δ*pik* **a** Δ*pap* **a** Δ*obp* **a** (Δ*nsg* **a**); all three of the NSGα alleles simultaneously, Δ*pik*α Δ*pap*α, Δ*obp*α (Δ*nsg*α); *PIK* **a** (Δ*pik* ***a***); *PIKα* (Δ*pikα*); *PAP* **a** (Δ*pap* ***a***); *PAPα*; (Δ*papα*); *OBP* **a**(Δ*obp* **a**); *OBPα* (Δ*obpα*); and both the **a** and α *OBP* alleles, (Δ*obp* **a/**Δ*obp*α) (Figure 1D). All of the mutants formed colonies on nutrient agar after five days with average diameters similar to those of their respective parent strains (data not shown). All of the mutants of both strains generated in strain P37039 exhibited growth kinetics in suspension cultures containing modified Lee's medium [29] that were indistinguishable from those of the parent strains (Figure 1 E). All of the mutants generated in strain P37039 also exhibited growth kinetics in suspension cultures containing the richer medium YPD [30] that were indistinguishable from those of the parent strain (Figure 1F). Changes in biofilm formation or virulence by any of the mutants generated for this study could, therefore, not be attributed to a major growth defect.

### Deletion of the a or α copy of the *MTL* locus affects biofilm formation

**Figure 2:**
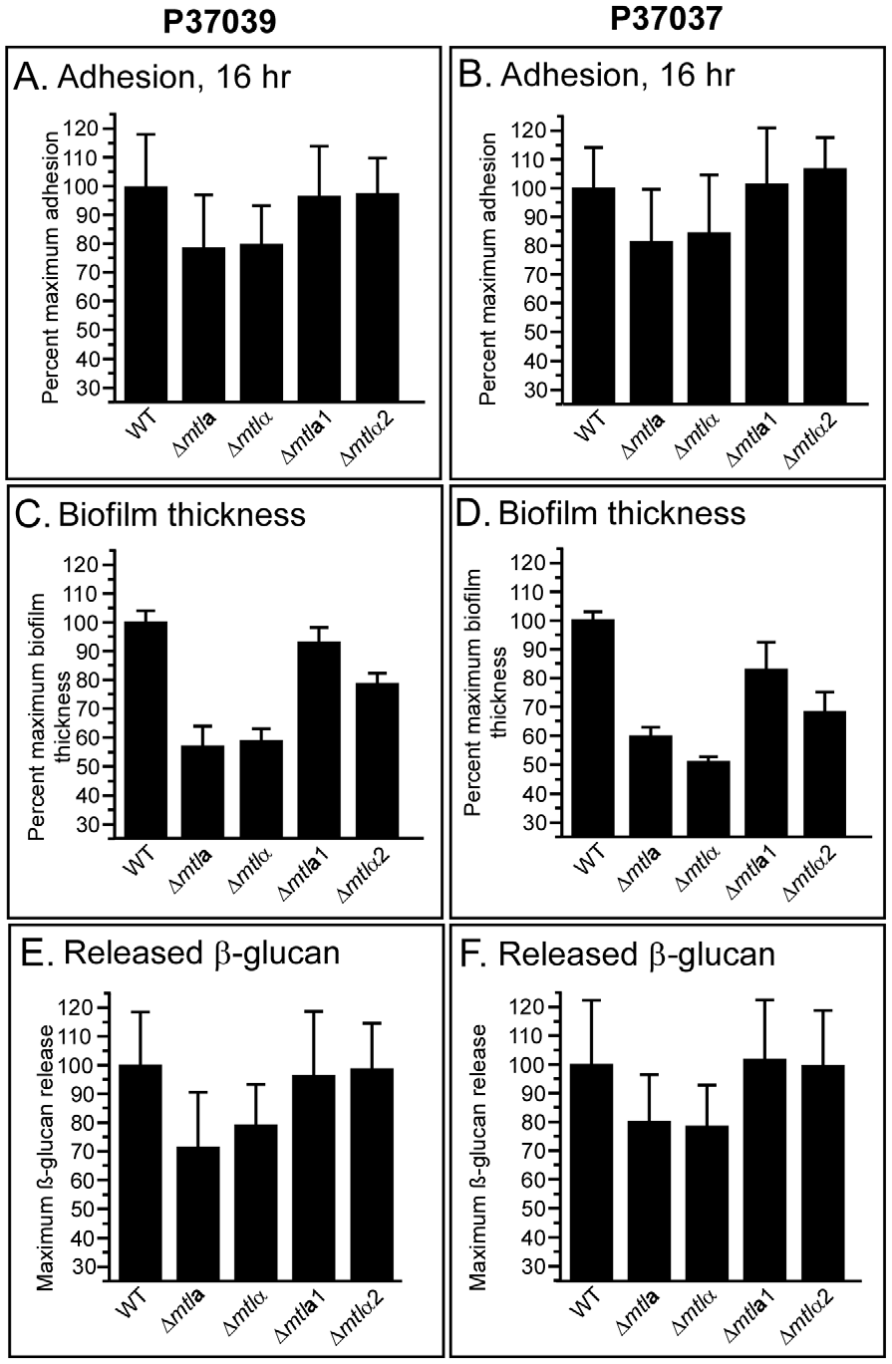
Deletion of the a or α copy of the *MTL* locus caused decreases in three biofilm parameters. Deletion of *MTL* **a**1 or *MTL*α2 caused decreases only in biofilm thickness. A, B. Cell adhesion to a plastic surface after 16 hours of incubation. C, D. Biofilm thickness after 48 hours. E, F. The release of β-glucan by biofilms after 48 hours.

Deletion of either the entire *MTL* **a** locus (Δ*mtl* **a**), which includes the genes *MTL* ***a*** *1, MTL* ***a*** *2, PIK* ***a*** *, PAP* ***a*** and *OBP* ***a***, or deletion of the entire *MTL*α locus (*Δmtl*α), which includes the genes *MTLα1, MTLα2, PIKα, PAPα* and *OBPα*, resulted in reductions in the three assessed characteristics of biofilm formation. These characteristics included adhesion to a plastic surface after 16 hours of incubation [31] (Figure 2 A, B), thickness of the biofilm that developed on a silicone elastomer surface after 48 hours [31] (Figure 2 C, D), and the level of β-glucan released from biofilms after 48 hour [31], [32] (Figure 2E, F). The decreases in thickness were significant (all p values were<2×10^4^) for the *Δmtl* **a** and *Δmtl*α mutants generated in each of the two strains. The decreases for adhesion were not significant (p values ranged from 0.18 to 0.068), and those for the release of β-glucan close to significant or significant (p values ranged from 0.081 to 0.043). However, the decreases were reproducible within a mutant strain and between mutant strains generated in different natural strains. The differences were consistent with those previously reported between the biofilms formed by parental strains and spontaneous **a/a** or α/α derivatives [33]. Deletion of either the **a** or α copy of *MTL*, however, had no discernible effect on the general architecture of biofilms (data not shown). Laser scanning confocal microscopy (LSCM) and scanning electron (SEM) revealed that after 48 hours, mutant biofilms, like parental P37039 and P37037 biofilms, possessed a basal layer of yeast cells at the substratum and an upper region of vertically oriented hyphae embedded in an acellular matrix (data not shown).

### Deletion of *MTL*a1 or *MTL*α2 affects only biofilm thickness

We next tested whether deletion of either of the two genes encoding the **a**1-α2 corepressor of switching and mating, *MTL* **a**1 or *MTL*α2, affected biofilm formation. Deleting either of these genes in both strains P37039 and P37037 had no apparent effect on either adhesion (Figure 2A, B) or the release of β-glucan (Figure 2E, F). The differences between wild type and mutants were statistically undistinguished (p values were close to 1.0). However, deleting either caused decreases in biofilm thickness (Figure 2C, D) that were significant (p values ranged from 6×10^−4^ to 10^−4^). These differences were obtained in three independent experiments performed for each of the two parental strains and mutant derivatives, measuring three locations in each of three biofilms of each strain. This provided 27 individual measurements per mutant or parental strain. In every experiment, the thickness of the biofilms formed by the *Δmtl* **a**1 mutant derivative was 10 to 20% less than that of the parental strain and the biofilm of the *Δmtlα*2 mutant 20 to 40% less than that of the parental strain (data not shown). LSCM and SEM revealed that the thinner biofilms formed by the *Δmtl* **a**1 and *Δmtl*α2 derivatives still exhibited the basic cell architecture of parental **a**/α biofilms, including a basal yeast cell layer and a larger upper portion containing vertically oriented hyphae interspersed with an acellular matrix (data not shown).

### Simultaneous deletion of all three NSGa or NSGα alleles affects biofilm formation

**Figure 3:**
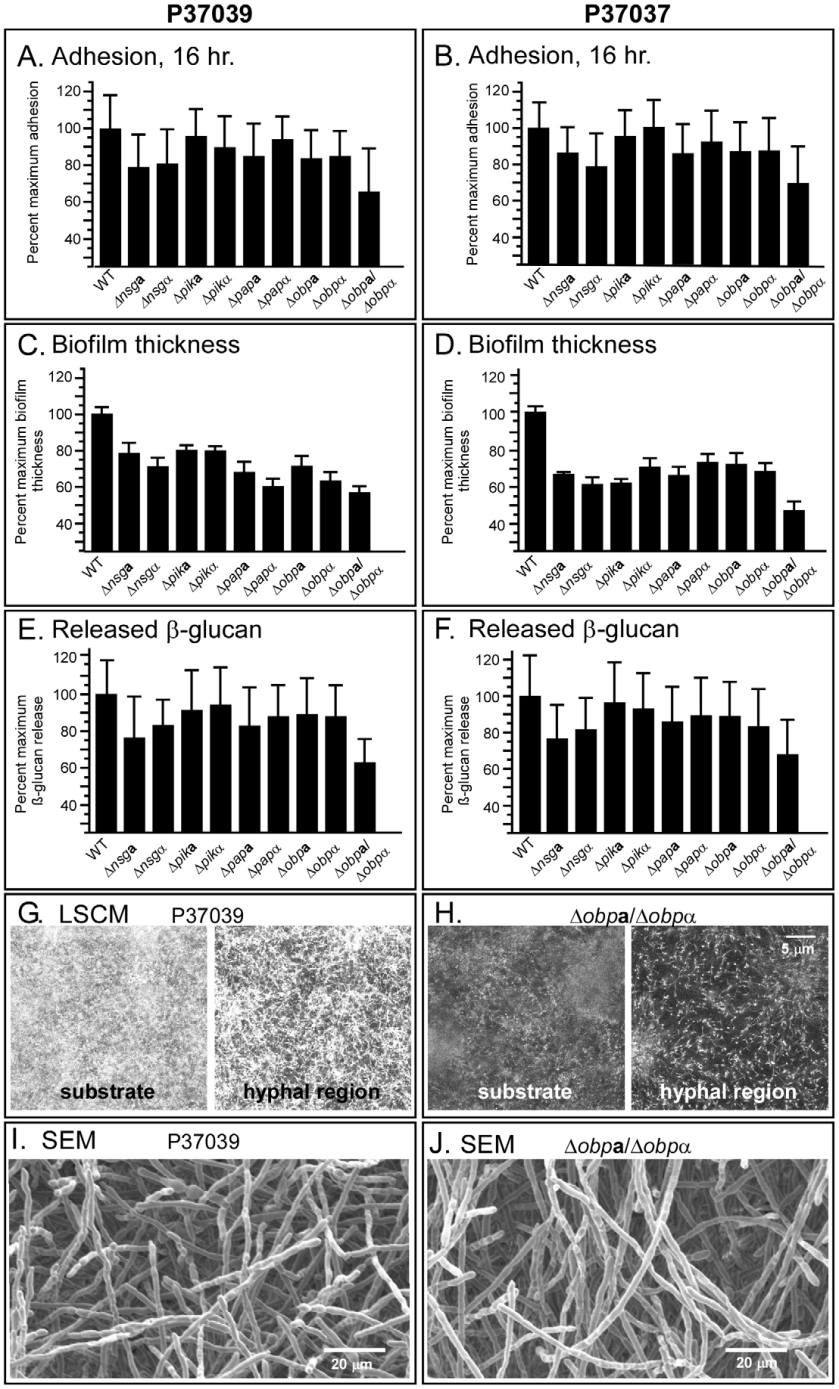
Deletion of the NSGs affected cell adhesion, biofilm thickness, β-glucan release and cell density. A, B. Cell adhesion to a plastic surface after 16 hours. C, D. Biofilm thickness after 48 hours. E, F. Release of β-glucan after 48 hours. G, H. Laser scanning confocal microscopy (LSCM) and morphology of P37039 wild type biofilms I, J. Scanning electron microscopy of the upper portion of fixed parental P37039 and Δ*obp* **a**/Δ*obp*α biofilms.

Deletion of *MTL* **a**1 and *MTL*α2, therefore, did not affect adhesion or release of β-glucan, and the decreases in the thickness of the biofilms formed by Δ*mtl* **a**1 and Δ*mtl*α2 cells, although significant, were not as great as those that occurred when the entire **a** or α locus was deleted in the mutants *Δmtl* **a** or *Δmtl*α, respectively (Figure 2C, D). These results suggested that it may have been the deletion of the NSG**a**s or the NSGαs in the full *MTL* deletion derivatives Δ*mtl*a and Δ*mtl*α that was responsible for the decreases in adhesion and β-glucan release, and a portion of the decreases in thickness observed in the full locus deletions. To test this directly, we generated the triple deletion mutant Δ*nsg* **a,** in which *PIK* **a**, *PAP* **a** and *OBP* **a** were deleted (*Δpik* **a** *Δpap* **a** *Δobp* **a**) and the triple deletion mutant Δnsgα, in which *PIKα, PAPα*, and *OBPα* were deleted (*Δpik*α *Δpap*α *Δobp*α), in both the natural strain P37039 and P37037 (Figure 1D). The homozygous triple deletion of the NSG**a**s and the NSGαs was not possible because both *PIK* and *PAP* are essential genes [13], [19]. The deletion mutants Δ*nsg* **a** and Δ*nsgα* generated from both test strains exhibited reductions in adhesion (Figure 3A, B), biofilm thickness (Figure 3C, D), and the release of β-glucan (Figure 3E, F). The reductions in adhesion and in the release of β-glucan were similar to those observed for the deletion mutants of the entire **a** or α copy of the *MTL* locus, *Δmtl* ***a*** and *Δmtl*α (Figure 2A, B and E, F, respectively). As was the case for the mutants Δmtla and Δmtlα, the p values for reductions in adhesion and the release of β-glucan for the *nsg* **a** and *nsg*α mutants were close to significant or significant (p values ranged from 0.05 to 0.23 for adhesion and 0.04 to 0.13 for the release of β-glucan. However, the decreases were reproducible within a mutant and between mutants generated in different natural strains. The decreases in thickness, however, for the two *nsg* **a** and *nsg*α mutants were highly significant (p values ranged from 3×10^−3^ to 2×10^−4^). The reductions in thickness were, however, highly significant with p values ranging from 10^−4^ to 6×10^−4^. The decrcreases in thickness for the *nsg* **a** and *nsg*α mutants were less pronounced than those for the *Δmtl* **a** or *Δmtl*α mutants (Figure 3C, D). That was consistent with the conclusion that *Mtla1* and *Mtlα2* were each partially responsible for biofilm thickness (Figure 2A and B). LSCM and SEM revealed normal general architecture for both *Δnsg* **a** and *Δnsg*α biofilm (data not shown).

### Deletions of individual NSGs affects biofilm formation

To assess which of the three NSGs played a role in biofilm formation, we deleted either the **a** or α copy of each NSG individually in the two strains P37039 and P37037. In both strains, the deletion mutant of either the **a** or α allele of each of the three NSGs resulted in a small or negligible decrease in adhesion (Figure 3A, B) and a small or negligible decrease in the release of β-glucan (Figure 3E, F). Although the consistency of the decreases in both strains suggested a trend, all of the decreases in adhesion and release of β-glucan for the individual NSG allelic mutants were insignificant (p value>0.05). All of the mutants, however, exhibited decreases in thickness (Figure 3C, D) that were significant (p values ranged between 10^−3^ and <10^−4^). Together, these results demonstrate that each of the **a** and α alleles of the NSGs play roles in **a**/α biofilm formation, most notably in biofilm thickness. Because the two mutants for each NSG allele were generated independently in two unrelated strains and exhibited similar defects, we considered complementation tests on all 26 mutants to be unnecessary. We therefore performed complementation tests on only two, Δ*pik* **a** and Δ*pap* **a** of strain P37039. This was accomplished by adding back wild type *PIK* **a** or *PAP* **a**, respectively, under the regulation of the *TET* promoter, to generate strains Δ*pik* **a**-*TET*p-*PIK* **a** and Δ*pap* **a**-*TET*p-*PAP* **a**. In both cases, addition, of 50 µg per ml of doxycycline, an inducer of the tetracycline promoter, completely, rescued the thickness defect (supplemental Figure S1).

### The *Δobpa/Δobp*α mutant exhibits severe defects in biofilm formation

Both *PIK* and *PAP* are essential genes [13], [19], so simultaneously deleting both the **a** and α copy of each to produce a homozygous deletion mutant was unattainable. Because *OBP* is not an essential gene [17], [21], we were able to generate the homozygous deletion mutant *Δobp* ***a*** */Δobp*α in both strain P37039 and P37037. The homozygous deletion mutant *Δobp* **a**/*Δobp*α of both strains exhibited far more dramatic decreases in adhesion (Figure 3A, B), biofilm thickness (Figure 3C, D) and release of β-glucan (Figure 3E, F) than either of the heterozygous *OBP* deletion mutants, *Δobp* **a** or *Δobpα,* the heterozygous deletion mutants of the other two NSGs (*Δpik* **a**, *Δpik*α, *Δpap*a and *Δpap*α), the deletion mutants for all three **a** or α copies of the NSGs (*Δnsg* ***a*** and *Δnsg*α) and the deletion mutants of the **a** or α copy of the *MTL* locus, (*Δmtl* **a** and *Δmtl*α). The decreases in adhesion were clost to significant or significant (p values were 0.06 and 0.01, respectively) and decreases in the release of β-glucan significant (p value were 0.01 and 0.002) The decreases in biofilm thickness were highly significant (both p values were <10^−4^).

The biofilms that were formed by the mutant *Δobp* ***a*** */Δobpα*, however, still retained the general architecture of parental wild type biofilms. LSCM of *Δobp* **a**/*Δobp*α biofilms revealed that they possessed a basal layer of yeast cells just above the substratum (Figure 3H) and vertically oriented hyphae 20 µm above the substrate (Figure 3H), similar to that of the parental strains (Figure 3G). The cell density of the mutants at the substratum and 20 µm above it, however, was lower than that of wild type biofilms (compare Figure 3G and H).

SEM revealed no differences between biofilms formed by the two parental strains and the deletion mutants *Δmtl* **a**, *Δmtl*α, *Δnsg* **a**, *Δnsg*α, *Δpik* **a**, *Δpik*α, *Δpap* **a**, *Δpap*α, *Δobp* **a** and *Δobp*α (data not shown). There was, however, a clear difference between the biofilms of the parental strain and that of the mutant *Δobp* **a**/*Δobp*α. *Δobp* **a**/*Δobp*α mutant biofilms had far less densely packed hyphae resulting in noticeable gaps in the upper hyphal region (Figure 3J). Biofilms of the parental strains lacked these large gaps (Figure 3I). Together, these results demonstrate that although deletion of both alleles of *OBP* had no effect on growth in liquid medium (Figure 1E) or on the general architecture of the biofilms they produced, it did have dramatic effects on early adhesion, thickness, the release of β-glucan (Figure 3A through F), and the density of hyphae in the upper regions of the biofilm (Figure 3G, H and I, J).

### Biofilm permeability and fluconazole susceptibility

**Figure 4:**
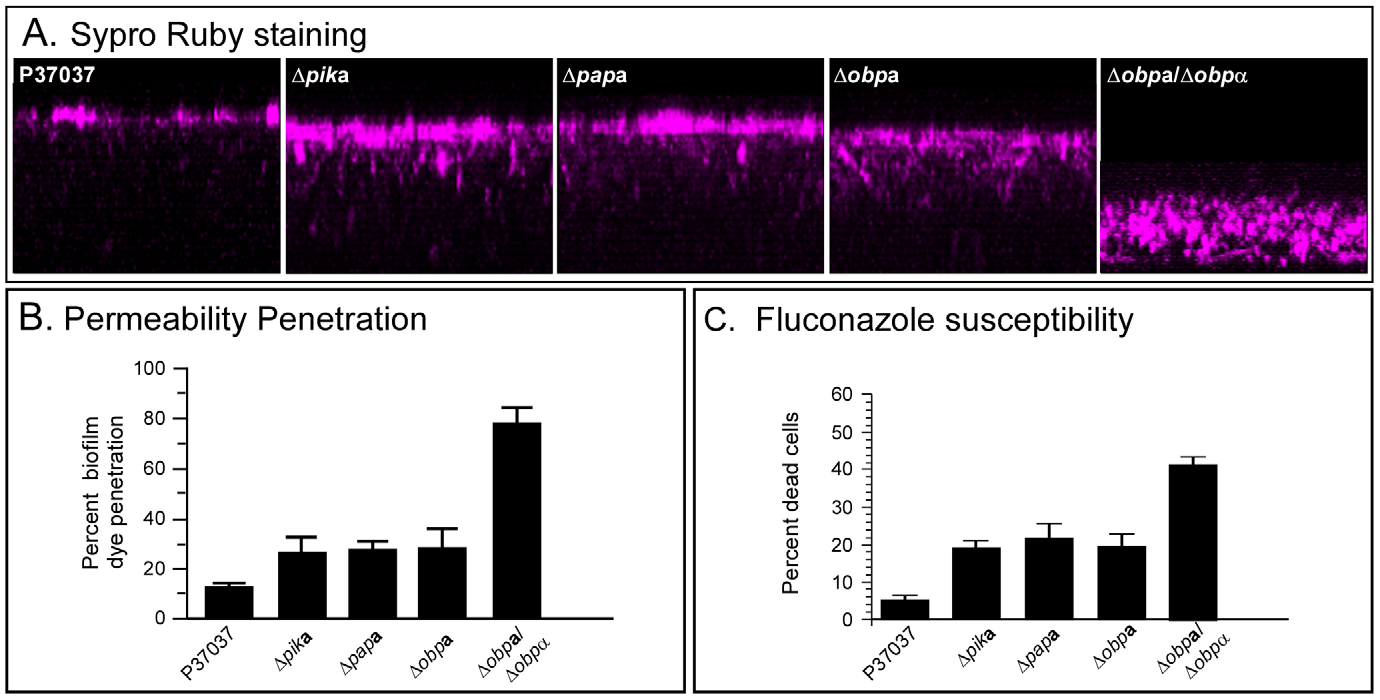
The biofilms formed by the mutants *Δpik*a, *Δpap*a and *Δobp*a show small increases in SYPRO Ruby permeability and fluconazole susceptibility, but the biofilms formed by the mutant Δ*obp*a/Δ*obp*α show larger increases in SYPRO Ruby permeability and fluconazole susceptibility. A. SYPRO Ruby penetration. B. Quantitation of SYPRO Ruby penetration. C. Fluconazole susceptibility.

Recently we demonstrated that biofilms formed by **a/**α strains of *C. albicans* were relatively impermeable to low and high molecular weight molecules, impenetrable by polymorphonuclear leukocytes and resistant to fluconazole [33]. In marked contrast, **a/a** and α/α biofilms were permeable to low and high molecular weight molecules, readily penetrated by polymorphonuclear leukocytes and susceptible to fluconazole [33]. We therefore first tested whether the biofilms formed by any of the NSG mutants generated in the **a**/α strain P37037 became more permeable to Sypro Ruby, which has a molecular weight of 1.6 kDa [34], [35]. The biofilms formed by the mutant derivatives *Δnsg* **a**, *Δpik* **a**, *Δpap* **a** and *Δobp* **a** were all slightly more permeable to Sypro Ruby than biofilms of the parental strains (Figure 4A, B). Percent penetration into the biofilm of the parental wild type strain P37037 was approximately 12%, while that of Δ*pik* **a**, Δ*pap* **a** and were approximately 28% (Figure 4A, B). The percent penetration of *Δobp* **a**/*Δobp*α biofilms, however, averaged approximately 80% (Figure 4A, B). We then tested resistance to fluconazole. While approximately 5% of cells in parental (P37037) biofilms were susceptible to fluconazole, approximately 20% of cells in biofilms of the mutants *Δpik* **a**, *Δpap* **a** and *Δobp*α were susceptible (Figure 4C). In marked contrast approximately 40% of cells in biofilms of *Δobp* **a**/*Δobp*α were susceptible (Figure 4C). These results indicate that *PIK, PAP* and *OBP* play roles in **a**/α biofilm impermeability and fluconazole resistance.

### The *Δobp*a/*Δobp*α mutant mates normally

**Figure 5:**
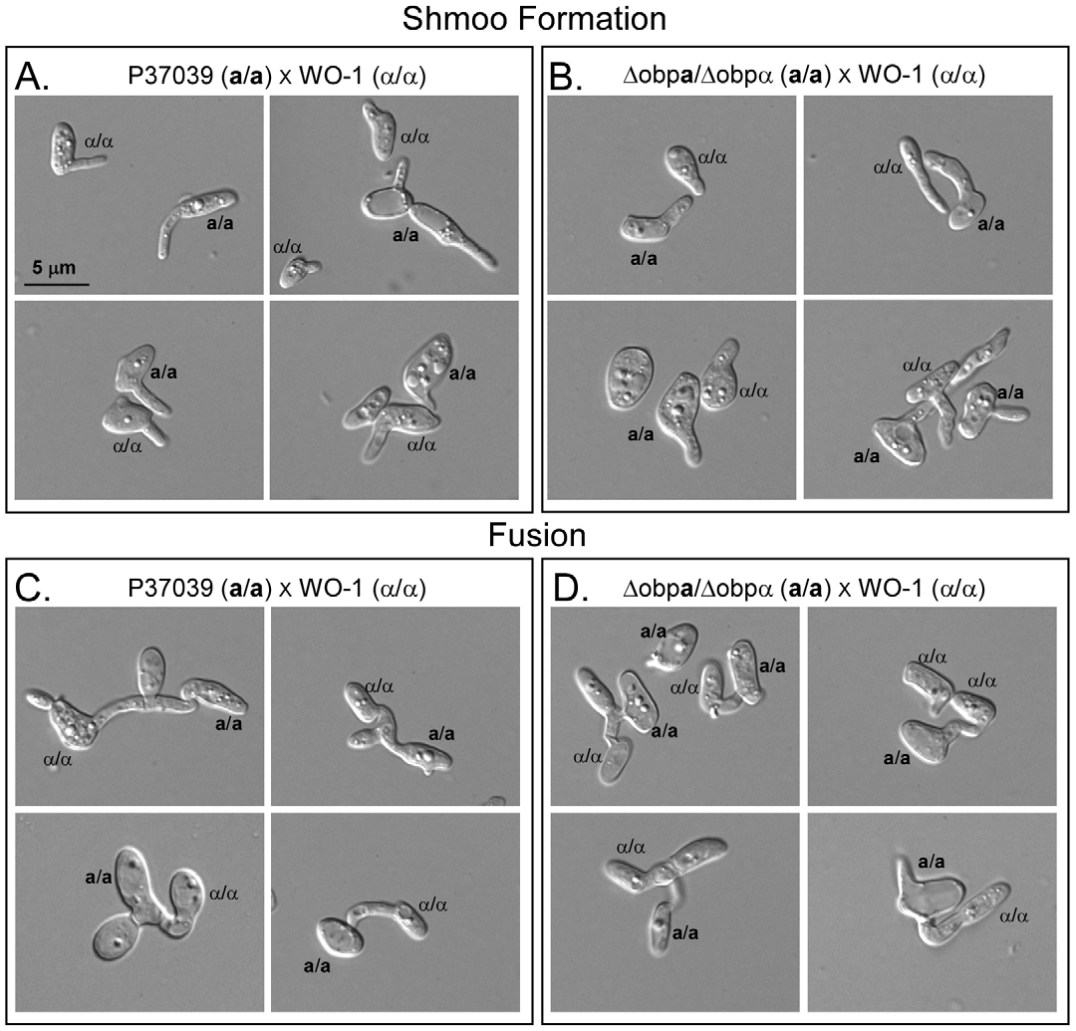
The homozygous *OBP* deletion mutant Δ*obp*a/Δ*obp*α mates normally. A. Shmoo formation in a cross between an **a/a** derivative of P37039 and α/α WO-1 cells. B. Shmoo formation in a cross between **a/a** Δ*obp* **a**/Δ*obp*α and α/α WO-1. C. Fusion between **a/a** P37039 and α/α WO-1. D. Fusion between **a/a** Δ*obp* **a**/Δ*obp*α and α/α WO-1. All crosses were done between opaque cells.

When NSGs are embedded in a mating type locus, there is always the question of whether they play a role in the mating process. Because only *OBP* was nonessential for growth, we were only able to test whether an **a/a** derivative of the homozygous *OBP* deletion mutant *Δobp* **a**/*Δobp*α could mate with an α/α strain, in this case strain WO-1 [36], [37]. **a/a** derivatives were generated by screening colonies of the **a**/α homozygous mutant *Δobp* **a**/*Δobp*α grown on sorbose medium for opaque colonies[38], [39], which reflect *MTL* homozygosis, and then by testing for the **a/a** genotype by polymerase chain reaction[38], [39]. Opaque cells of the **a/a** derivatives of Δ*obp* **a**/Δ*obp*α mated with opaque cells of the α/α strain WO-1 (Figure 5). Both the formation of evaginated cells (shmoos) and fusion occurred normally (Figure 5). These results demonstrate that at least one of the NSGs, the nonessential gene *OBP*is not required for shmoo formation or fusion in the mating process.

### Overexpression of *PIK* and *PAP* partially rescues *Δ*obpa/*Δ*obpα defects

The decreases in adhesion and the release of β-glucan, the dramatic decrease in thickness, the decreases in hyphal density, and the loss of impermeability and drug resistance by the Δ*obp* **a**/Δ*obp*α mutant, demonstrate that *OBP* plays a major role in **a**/α biofilm development. But because we could not generate homozygous *PIK* and *PAP* deletion mutants due to the essential roles they play in growth [9], [13], we were unable to distinguish if *OBP* alone or if all three of the NSGs were necessary for the above biofilm characteristics. To explore this question, we used an overexpression strategy. If overexpression of *PIK* or *PAP* rescued the *Δobp* **a**/*Δobp*α defects in biofilm formation, it would answer two questions, first whether *PIK* or *PAP* contributed to **a**/α biofilm permeability and fluconazole resistance, and second, whether over-expression of one NSG could rescue the defects of the homozygous deletion of another. We therefore placed wild type *PIK* or *PAP* under the regulation of the *TET* promoter, and inserted the construct into one allele of the gene *ADH1* [40] in the P37039 mutant *Δobp* **a**/*Δobp*α. Overexpression of the inserted gene was achieved by adding 50 µg per ml of doxycycline at 0 hr followed after 24 hr by addition of 25 µg per ml during biofilm development. Overexpression wild type *PIK* **a** returned biofilm thickness to that of wild type, and overexpression of *PAP* **a** to a thickness greater than wild type (supplemental Figure S1A). Overexpression, however, only partially rescued impermeability to Sypro Ruby and fluconazole resistance (supplemental Figure S1B and C, respectively). Sypro Ruby penetrated into 5% in P37039 biofilms, but into approximately 80% of Δ*obp* **a**/Δ*obp*α biofilms (supplemental Figure S1B). It penetrated into approximately 55% of Δ*obp* **a**/Δ*obp*α-*TET*p-*PIK* **a** biofilms and 50% of Δ*obp* **a**/Δ*obp*α-*TET*p-*PAP* **a** biofilms (supplementary Figure S1B). Cell death due to fluconazole treatment was approximately 4% in P37039 biofilms, but approximately 43% in Δ*obp* **a**/Δ*obp*α biofilms. It was approximately 25% in Δ*obp* **a**/Δ*obp*α-*TET*p-*PIK* **a** biofilms and 20% in Δ*obp* **a**/Δ*obp*α-*TET*p-*PAP* **a** biofilms (supplemental S1C). Therefore, overexpression of either *PIK* **a** or *PAP* **a** in a *Δobp* **a**/*Δobp*α background completely rescued the thickness defect, but only partially rescued the permeability and fluconazole susceptibility defects.

### *MTL*a1, *MTL*α2, and the three NSGs are constitutively expressed

**Figure 6:**
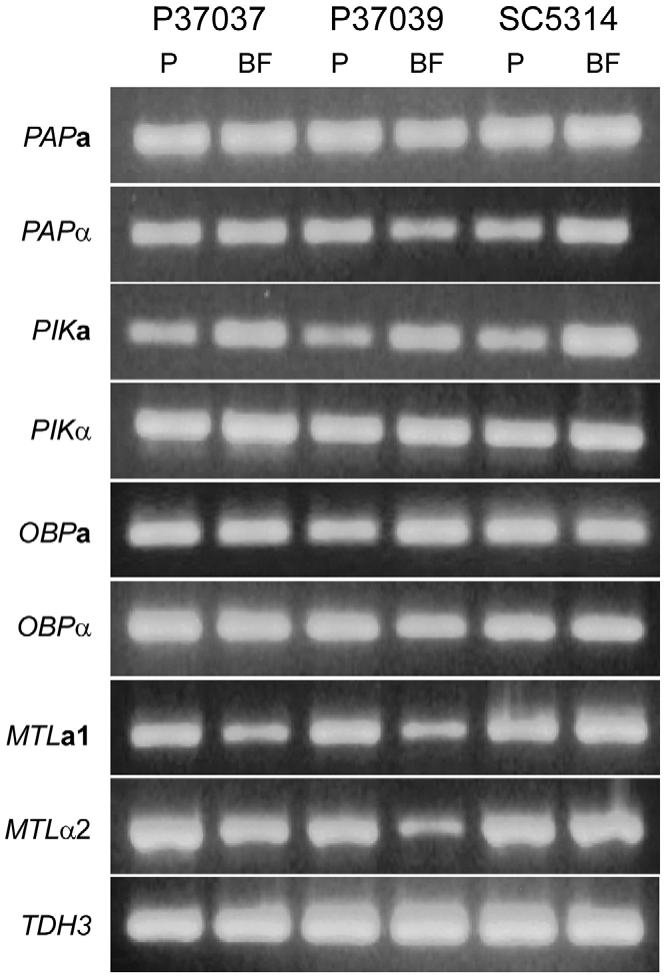
Both the NSGs and the mating genes *MTL*a1 and *MTL*α2 are constitutively and similarly expressed during planktonic growth (P) and biofilm formation (BF). Analyses were performed in three unrelated a/α strains, P37037, P37039 and SC5314. RT-PCR was used to assess expression. The constitutively expressed gene *TDH3* was analyzed as a control.

Using RT-PCR, we compared the expression levels in the natural strains P37037 and P37039, and the laboratory strain SC5314, of *MTL* **a**1, *MTL*α2, and each allele of the three NSGs. Measurements were made after 14 hours of planktonic growth or biofilm formation. *MTL* **a**1 and *MTL*α2, each of the two alleles of the three NSGs were expressed both during planktonic growth and biofilm formation (Figure 6). The only difference consistent among the three test strains was expression of the *PIK* **a** transcript, which showed higher levels in biofilms (Figure 6). Control experiments using RT-PCR to assess expression in mutants verified that the probes used were specific for the mating type genes and NSG alleles.

### Virulence in a mouse systemic model

**Figure 7:**
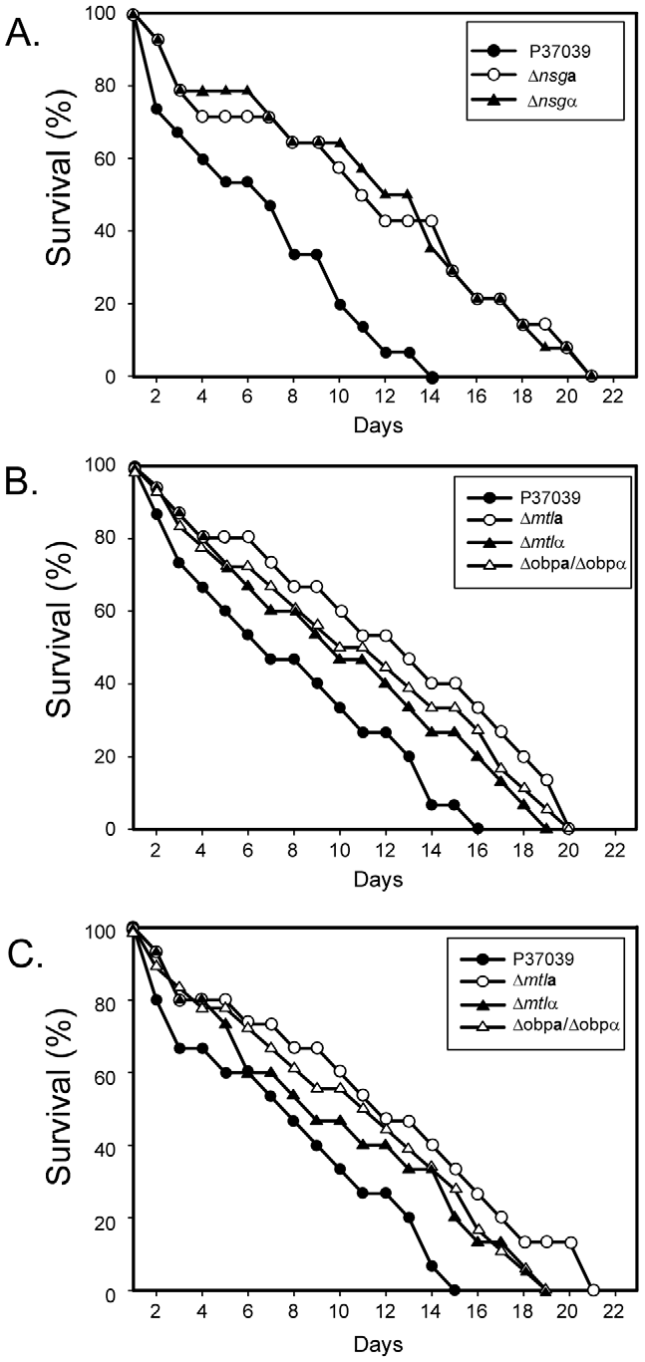
Deletion of either copy of the mating type locus, *MTL*a or *MTL*α, of either the NSGas or the NSGαs, or the two OBPs, *OBP*a and *OBP*α, caused a decrease in virulence of the a/α strain P37039 in the mouse tail injection model for systemic infection. Fifteen mice were injected with each noted strain. A, B and C represent three separate experiments in which injected mice were monitored over a 22 day period. Uninjected control mice exhibited 0 to 7% death after 22 days.

Finally, we compared the virulence of the select mutants Δ*mtl* **a**, Δ*mtl*α, Δ*nsg* **a**, Δ*nsg*α and Δ*obp* **a/**Δ*obp*α with the parental strain P37039, using the mouse tail injection model for systemic infection [26], [41]. Host survival increased for every tested deletion mutant (Figure 7A, B, C). The differences in survival, computed by the log rank test for survival, were significant between P37039 and the derivative mutants *Δnsg* **a** (p value 0.008) and *Δnsg*α (p value 0.005) (Figure 7A). The differences in survival were significant, for the pooled data of the repeat experiments (Figure 7B and C), between P37039 and *Δmtl* **a** (p value 0.003), between P37039 and *Δmtlα* (p value 0.007) and between P37039 and *Δobp* **a** */Δobp*α (p value 0.006). These results indicate that in addition to **a**/α biofilm formation, the *MTL* locus and at least one specific NSG embedded in it, *OBP*, play roles in virulence in the mouse model for systemic infection.

## Discussion

The majority of *C. albicans* biofilms that form on catheters, prosthetics and tissues are due to strains that are heterozygous (**a**/α) at the mating type locus *MTL* [42]–[44]. Such biofilms are thick, impermeable to molecules ranging in molecular weight from 300 to 140,000 daltons, impenetrable by leukocytes and resistant to fluconazole [33]. The developmental program for the formation of these biofilms is complex, resulting in a multicellular structure containing multiple phenotypes embedded in a multimolecular, extracellular matrix. The film is more than 50 times thicker than a yeast cell monolayer. These **a**/α biofilms are composed of a thin basal layer of highly adherent and cohesive yeast cells, and a thick upper layer of vertically oriented hyphae, originally formed from the yeast cells in the basal layer. These intertwined hyphae are embedded in a multimolecular matrix produced by the cells in the developing biofilm [45]–[48]. At the upper edge of a biofilm, yeast cells are formed by the hyphae, which appear to function as a dispersal mechanism [45], [49], [50]. **a**/α biofilms are regulated by the Ras1/cAMP pathway, which targets a transcription factor cascade consisting of Efg1→Tec1→Bcr1 [45], [49], [50]. Because the majority of biofilms formed in nature must be **a**/α, elucidating the molecular mechanisms that mediate their resistance to antifungals [33], [50], [51], their impermeability to molecules in the molecular weight range of antibodies, and their capacity to exclude polymorphonuclear leukocytes, will facilitate therapeutic strategies for this pervasive human pathogen.

Here we have investigated whether the nonsex genes embedded in the mating type locus play common or related nonmating roles in pathogenesis, in particular in the formation of **a**/α biofilms. We had previously presented evidence that natural strains that were homozygous at the *MTL* locus at the time of isolation, derivatives of natural **a**/α strains in which the **a** or α copy of *MTL* was specifically deleted and derivatives of natural **a**/α strains that had undergone spontaneous homozygosis *in vitro* at the *MTL* locus, were on average less virulent in the mouse tail-injection model for systemic infection than **a**/α strains [26]. In addition, we had demonstrated that although **a/a** or α/α cells biofilms were architecturally similar to **a**/α biofilms, they were thinner, far more permeable to low and high molecular weight molecules, far more susceptible to fluconazole, and readily penetrated by polymorphonuclear leukocytes. Therefore, only **a**/α biofilms exhibit those traits one would expect of a pathogenic biofilm. Moreover, *MTL-* homozygous biofilms were found to be regulated by the MAP kinase pathway, in contrast to **a**/α biofilms, which are regulated by the Ras1/cAMP pathway [33]. Together, these results suggested to us that the genes in the *MTL* locus, including the NSGs, could play non-mating roles in the formation of pathogenic **a**/α biofilms and in virulence in the mouse model for systemic infection.

### The roles of the *MTL* genes in adhesion, thickness and β-glucan release

One might assume that one or more of the NSGs may play roles in mating, as was suggested as a possibility by Fraser *et al*. [8] for nonessential genes embedded in the *MAT* locus of *Cryptococcus*. In *C. albicans*, we have, however, found that this is not the case for the single nonessential NSG, *OBP*. The homozygous deletion of *OBP* in an **a/a** background had no effect on α-pheromone-induced evagination (“shmoo” formation) or on fusion with cells of the opposite mating type.

Deleting simultaneously all of the **a** or the α alleles of the NSGs did, however, had an effect on six **a**/α biofilm parameters. Individual allelic deletions of NSGs caused significant decreases in biofilm thickness, but had very limited effects on adhesion and β-glucan synthesis. Decreases in these two parameters were not statistically significant within an experiment, but were reproducible between experiments for a mutant of a single strain and among experiments for the two mutants of the unrelated parental strains.

Deleting either of the two mating type genes, *MTL* **a**1 or *MTL*α2, also caused a significant decrease in biofilm thickness, but had no discernible effect on adhesion or the release of β-glucan. The differential effects of the mating type genes and NSGs on biofilm parameters was not surprising given that each of the three parameters represented a very different and inherently complex aspect of biofilm formation. First, the increase in adhesion to a silicon elastomer surface, measured after sixteen hours of biofilm development, is a response by the initial yeast cell inoculum and their immediate yeast cell offsprings [31]. No hyphae have formed at the time of that assay. Thickness on the other hand is measured after 48 hours of biofilm development and reflects the extent of vertical hyphal elongation supported by extracellular matrix [52], [53]. The release of β-glucan is also measured after 48 hours and has been interpreted as a reflection of matrix deposition [54]–[56]. Permeability is assessed after 48 hours of biofilm development by adding a solution of Sypro Ruby to the top of a living biofilm and then measuring how deep the dye has penetrated after 30 additional minutes [33]. The cell death assay is a similar measure of fluconazole penetration [33]. And finally, we assessed the presence of a basal yeast cell layer, hyphal orientation and hyphal density in 48 hour biofilms using LSCM and SEM. Our results demonstrate that *MTL* **a**1 and *MTL*α2 play selective roles in biofilm thickness, but not in adhesion or the release of β-glucan, whereas the NSGs may play roles in all of these biofilm traits. The observation that overexpression of *PIK* or *PAP* fully rescued the thickness defect of Δ*obp* **a**/Δ*obp*α biofilms, but only partially rescued the permeability and fluconazole susceptibility defects again dissociates biofilm parameters. These results underscore the complexity of biofilm formation and the dissociability of traits, and therefore the need to use multiple and independent measures of the different aspects of biofilm development in analyzing each mutant phenotype. Incomplete rescue by overexpressing PIK or PAP suggests again that the roles of the NSGs are not simply additive, a reasonable expectation given the disperate functions of the three NSGs.

The Δ*obp* **a**/Δ*obp*α mutants were defective in all biofilm parameters, and the decreases in these parameters were approximately double those measured for any of the heterozygous *MTL* deletion mutants or for the *MTL* **a**1 and *MTL*α2 deletion mutants. Moreover, the gaps in the hyphal network in the upper regions of Δ*obp* **a**/Δ*obp*α biofilms were not observed in biofilms formed by the Δ*mtl* **a**1, Δ*mtl*α2 or individual allelic NSG deletion mutants. Yet the qualitative aspects of biofilm architecture, which included the general organization of cell types and matrix, was maintained in the *Δobp* **a**/Δ*obp*α mutant biofilm. It therefore appears that the mating type gene *MTL* **a**1 and *MTL*α2, and the NSGs PIK, PAP and OBP affect the quality of a biofilm rather than the basic structure.

### The role of NSGs in biofilm permeability and drug susceptibility

Two of the most important characteristics of a pathogenic biofilm are impermeability to such molecular challenges as antibodies [33], [57] and antifungals (i.e., drug resistance) [33], [58]–[62], and resistance to white blood cell penetration [33]. The **a**/α biofilm exhibits all of these characteristics, and for that reason has been distinguished as a “pathogenic” biofilm [33]. Here, we have shown that deleting one of the alleles of any of the three NSGs in an **a**/α strain has a small but reproducible effect on permeability and fluconazole susceptibility, and that deleting both alleles of *OBP* results in a major increase in both parameters. These results suggest that *OBP*, as well as PIK and PAP, play major roles in establishing the pathogenic traits of an **a**/α biofilm.

Total rescue of thickness and partial rescue of impermeability and fluconazole resistance in Δ*obp* **a**/Δ*obp*α **a/**α biofilms in which either *PIK* **a** or *PAP* **a** are overexpressed suggests at least partial additivity or roles in a dependent pathway. In the latter case, *PIK* and *PAP* may play roles downstream of *OBP*. Although the disperate functions of the mating type genes and the three NSGs do not provide any obvious explanation for the related roles the MTL genes play in biofilm formation, it is worth considering the known functions and regulations of NSG orthologs in other systems. *OBP*s encode a family of proteins involved in the transport and metabolism of sterols [17]–[21], [63]. They have also been shown to serve as regulators of genes involved in lipid metabolism and intracellular signaling [63]. *OBP* gene products not only bind oxysterols, but also phosphoinositides, presumably an interaction involved in the regulation of sterol binding [21]. *PIK* encodes a kinase involved in the first step in the production of inositol-1, 4, 5-triphosphate [64]. In *S. cerevisiae*, fractionation studies suggest that the *PIK* ortholog is involved in a nuclear phosphoinositide regulatory process [64]. The poly(A)polymerase, encoded by *PAP*, catalyzes 3′ polyadenylation of mRNAs, a necessary step in transport and stability [65], [66], in the nucleus [66], [67]. Hence, there are loose or indirect connections between the three NSGs in other organisms ranging in complexity from yeast to man. There have been, however, no reported experiments performed to date in *C. albicans* that could be used to explain the mutant and overexpression results functionally linking these three colocalized genes, and the two mating type genes *MTL* **a**1 and *MTL*α2, to the formation of **a**/α biofilms. Given the importance of **a**/α biofilms in commensalism and pathogenesis, elucidating such relationships would be highly relevant in the future.

### The role of NSGs in virulence in a mouse model

Finally, we tested whether the NSGs contributed to the role the MTL played in virulence in the mouse model for systemic infection [26]. Our results demonstrate that deleting MTLa or MTLα locus, the NSG**a**s or the NSGαs, or both copies of *OBP* results in similar decreases in virulence. Although it is not at all clear that biofilm formation plays any role in this model for systemic infection, it is clear that the *MTL* locus and the resident NSGs play a role not only in biofilm formation, but also in the virulence of **a**/α cells, two pathogenic traits apparently unrelated to mating.

## Materials and Methods

Animal care and usage was in accordance with the National Institutes of Health guidelines for the use of laboratory animals in biological research. Experimental protocols were reviewed by the Institutional Animal Care and use Committee (IACUC) at the University of Iowa (ACURF no. 1101003).

### Strains and media

The names, genotypes and origins of the basic strains and mutant derivatives used in this study are listed in Figure 1D, and the basic strains and mutants used for complementation, mating and overexpression experiments are listed in supplemental Table S1. Growth conditions were previously described [31], [36]. Supplemented Lee's medium [68] was employed for basic growth experiments. *MTL* genotypes of the parent strains were verified by PCR prior to use in experiments.

### Generating hemizygous mutants

All mutants were generated for this study using the pop-out flipper cassette from pSF52A (40) containing the dominant nourseothricin resistance marker *CaSAT1*. To increase the efficiency of deletion, the Sma1 sites flanking the *SAT*-flipper cassette were subcloned into the pGEM-T Easy, to generate pGEM2A. All of the primers used are listed in supplemental Table S1. The genotypes of the mutants employed in this study are presented in Figure 1D. For each hemizygous deletion mutant, the cassettes were constructed in two steps. First, the allele-specific 5′ and 3′ flanking fusion fragment was generated by PCR. Second, the SmaI-digested *SAT*-flipper cassette from pGEM2A was insented in between the flanking fragment. The deletion of the alleles of all three non-sex genes of *MTL* **a** was created in one-step due to their contiguous arrangement in the locus. Deletion of the NSG alleles of *MTLα* was carried out in two steps. First, two deletion cassettes were created, one for the *PAP* **a** allele and second, for the *OBP* **a**-*PIK* **a** allelic pair. Deletion cassettes were isolated from the plasmids by digestion with SacI and SphI, then used for transformation of parental strains. The putative transformants were verified by PCR. These transformants were also verified for **a**/α heterozygosity using the primers listed in supplemental Table S2.

### Strains for complementation and overexpression

To test for complementation of the mutants *Δpik* **a** and *Δpap* **a** of strain P37039, *PIK* **a** and *PAP* **a**, respectively were reintroduced under regulation of the *TET* promoter (40), using methods previously described in detail [33]. For overexpression of *PIK* **a** and *PAP* **a** in the mutant *Δobp* **a**/*Δobp*α, the same strategy was employed, using *ADH1* (40) as the insertion locus. The primers used to create the constructs are listed in supplemental Table S2.

### Generation of mating-competent strains

To test whether the *Δobp* **a**/*Δobp*α strain could mate, homozygous **a/a** derivatives of the P37039 mutant were generated using sorbose induction [38], [39]. The **a/a** configuration was verified by PCR. Shmoo formation and fusion in suspension cultures of the **a/a** derivative of *Δobp* **a**/*Δobp*α and the α/α strains WO-1 was performed according to methods previously described [37]. The **a/a** cells were stained with Alexa 488 (Invitrogen) and the α/α cells with TR/TC Con-A (Invitrogen) according to methods previously described [37].

### Measuring biofilm parameters

In brief, adhesion was measured by incubating cells in the wells of a 12 well plates for 16 hour, washing gently, then trypsinizing to release cells for counting [31]. Biofilm thickness was measured after 48 hours of biofilm development by Laser scanning confocal microscopy [31]. β-glucan measurements were done according to the methods of Mitchell and coworkers [32], [69] with supernatants of 48 hour biofilms. Measurements of Sypro Ruby permeability and fluconazole susceptibility have recently been described in detail [33]. In brief, 48 hour biofilms were overlaid with Biofilm Tracer Sypro Ruby (Invitrogen) for 30 minutes prior to laser scanning confocal microscopy, as previously described [33], using a Bio-Rad Radiance 2100 MP laser scanning confocal microscope (Bio-Rad, Hermel Hampstead, United Kingdom) equipped with a Mai-Tai IR laser (Spectra-Physics Lasers, Mountain View, CA). Calcofluor was imaged using the Mai-Tai IR titanium-sapphire laser tuned to 818nm. Fluconazole susceptibility was measured as previously described in detail [33]. In brief, 25 µg per ml of fluconazole was added to the top of 48 hr biofilms and incubated an additional 24 hours. Biofilms were disassociated in 20 µM EDTA and cells assessed for viability by Dead Red staining. Scanning electron microscopy was performed by fixing biofilms in 2.5% glutaraldehyde in 0.1M cacodylate buffer. After post-fixation in 1% osmium, the biofilms were dehydrated through a graded ethanol series and further dehydrated by 50% hexamethydisilazane (HDMS) followed by two rinses in 100% HDMS. The biofilms were allowed to air-dry prior to sputter-coating with a 60∶40 mixture of gold and palladium. The biofilms were viewed using a Hitachi S-4800 scanning electron microscope.

### RT-PCR

The methods used for RT-PCR have been described previously in detail [33], [69].

### Virulence in a systemic mouse model

The method used to assess the virulence has been described previously in detail [26]. Mice were examined every 12 hours. Mice exhibiting the first sign of morbidity were euthanized by inhalation of saturated CO_2_.

## Supporting Information

Figure S1 **Complementation and over-expression strategies.** A. Thickness of biofilms in P37039, Δ*pik* **a,** Δ*pap* **a,** Δ*obp* **a**/Δ*obp*α and the respective mutants in which *PIK* **a** and *PAP* **a** are expressed under the regulation of the *TET* promoter, (*TET*p). B. Sypro Ruby permeability of P37039, Δ*obp* **a**/Δ*obp*α and the latter mutant in which either *PIK* **a** or *PAP* **a** is overexpressed under the regulation of the *TET* promoter, *TET*p. C. Fluconazole susceptibility in the same strains analyzed in panel C.
(TIF)

Table S1 **Strains used in complementation, additivity and mating studies.**
(DOCX)

Table S2 **Oligonucleotides used in this study.**
(DOC)
