# Supplementary figures and images for: Nonsex Genes in the Mating Type Locus of Candida albicans Play Roles in a/α Biofilm Formation, Including Impermeability and Fluconazole Resistance
Source: PLoS Pathog. 2012 Jan 12;8(1):e1002476. doi: 10.1371/journal.ppat.1002476 (PMC3257300; doi:10.1371/journal.ppat.1002476)

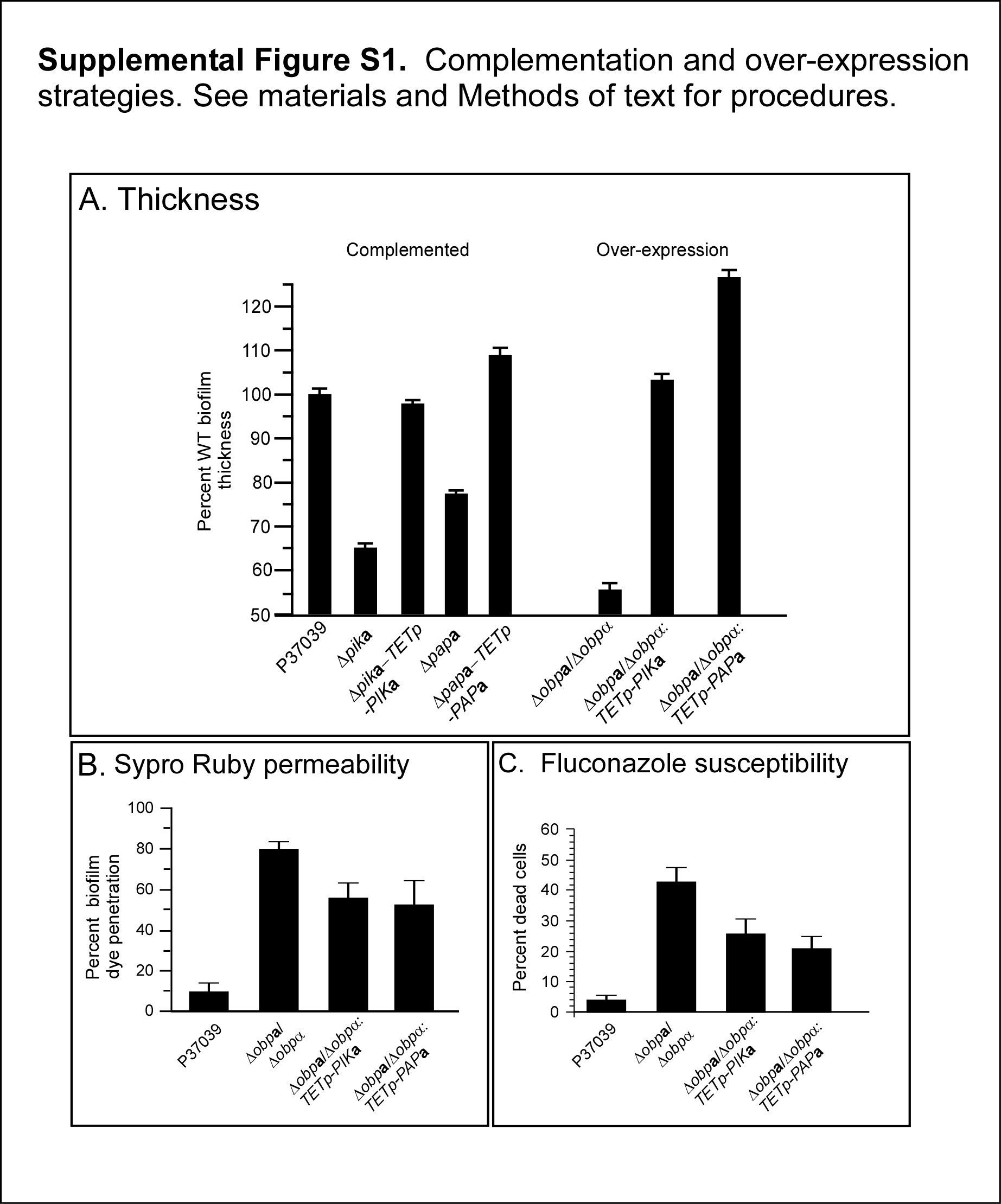

Supplement: Figure S1 — Complementation and over-expression strategies. A. Thickness of biofilms in P37039, Δpik a, Δpap a, Δobp a/Δobpα and the respective mutants in which PIK a and PAP a are expressed under the regulation of the TET promoter, (TETp). B. Sypro Ruby permeability of P37039, Δobp a/Δobpα and the latter mutant in which either PIK a or PAP a is overexpressed under the regulation of the TET promoter, TETp. C. Fluconazole susceptibility in the same strains analyzed in panel C. (TIF) [file ppat.1002476.s001.tif]
