# Supplementary material for: Nonsex Genes in the Mating Type Locus of Candida albicans Play Roles in a/α Biofilm Formation, Including Impermeability and Fluconazole Resistance
Source: PLoS Pathog. 2012 Jan 12;8(1):e1002476. doi: 10.1371/journal.ppat.1002476 (PMC3257300; doi:10.1371/journal.ppat.1002476)
Supplement: Table S1 — Strains used in complementation, additivity and mating studies. (DOCX) [file ppat.1002476.s002.docx]

**Supplemental Table S1. Strains used** in **complementation, additivity and mating studies.**

| Strains | Parent | MTL | | Genotype | Source |
| --- | --- | --- | --- | --- | --- |
|  |  |  | |  |  |
| *∆pap***a***-TET*p*-PAP****a*** | *∆pap***a** | **a**/α | | *∆pap***a***::FRT/PAP***a***..adh1::pTET-PAP***a***-GFP::NAT* | This study |
| *∆pik***a***-*TETp*-PIK****a*** | *∆pik***a** | **a**/α | | *∆pik***a***::FRT/PIK***a***..adh1::pTET-PAP***a***-GFP::NAT* | This study |
| *∆obp***a***/∆obpα-TET*p*-PAP****a*** | *∆obp***a***/∆obpα* | **a**/α | | *∆obp***a***::FRT/∆obpα::FRT..ADH1/adh1::pTET-PAP***a***-GFP::NAT* | This study |
| *∆obp****a****/∆obpα-TET*p*-PIK****a*** | *∆obp***a***/∆obpα* | **a**/α | | *∆obp***a***::FRT/∆obpα::FRT..ADH1/adh1::pTET-PIK***a***-GFP::NAT* | This study |
| P37039*(***a***/***a***)*  ∆*obp***a**/∆*obp****α*** | P3703  ∆*obp***a**/∆*obp*α | | ***a****/***a**  **a***/***a** | Sorbose induced homozygote  Sorbose induced homozygote | This study  This  study |
