# Supplementary material for: Nonsex Genes in the Mating Type Locus of Candida albicans Play Roles in a/α Biofilm Formation, Including Impermeability and Fluconazole Resistance
Source: PLoS Pathog. 2012 Jan 12;8(1):e1002476. doi: 10.1371/journal.ppat.1002476 (PMC3257300; doi:10.1371/journal.ppat.1002476)
Supplement: Table S2 — Oligonucleotides used in this study. (DOC) [file ppat.1002476.s003.doc]

|  | |  | |
| --- | --- | --- | --- |
|  |  |  | |
| **Supplemental Table S2. Oligonucleotides used in this study** | | | |
|  |  |  | |
| **Primer** | **Gene/Purpose** | | **Sequence** |
| obpaf1 | Deletion of OBPa allele | | 5’-TCGCTCGAGGTTCATGACTGTCGCATAGAACC-3’ |
| obpar1 | Deletion of OBPa allele | | 5’-TCGCCCGGGCTTGACTTCGTACTGAATAC-3’ |
| obpaf2 | Deletion of OBPa allele | | 5’-TCGCCCGGGGAAATACCAGCATTCAAGAAAC-3’ |
| obpar2 | Deletion of OBPa allele | | 5’-TCGCTCGAGGGTTCAGTGTATTGATGATGTA-3’ |
| oachkf2 | Verify OBPa deletion | | 5’-TGGTGAACACCTATGTTGTC-3’ |
| obpbf1 | Deletion of OBPα allele | | 5’-TCGCTCGAGGCTTGAACTATCCTTATACC-3’ |
| obpbr1 | Deletion of OBPα allele | | 5’-TCGCCCGGGTACTGATTACCTGAACCTTC-3’ |
| obpbf2 | Deletion of OBPα allele | | 5’-TCGCCCGGGGTTTCACCCTAAGTTCTTTC-3’ |
| obpbr2 | Deletion of OBPα allele | | 5’-TCGCTCGAGAGTTTATGATGATTACGTGGTCC-3’ |
| pochkf1 | Verify OBPα deletion | | 5’-TCGGCTCATGTTGAATCCCAATGC-3’ |
| papaf1 | Deletion of PAPa allele | | 5’-TCGCTCGAGCTTGACTTCGTACTGAATAC-3’ |
| papar1 | Deletion of PAPa allele | | 5’-TCGCCCGGGCTTGACTTCGTACTGAATAC-3’ |
| papaf2 | Deletion of PAPa allele | | 5’-TCGCCCGGGATTTATAGAAGGTTCCTAGGGTAAC-3’ |
| papar2 | Deletion of PAPa allele | | 5’-TCGCTCGAGGTTGGCCATAAATTATACAC-3’ |
| apchkf1 | Verify PAPa deletion | | 5’-TTGGTGATTCAATTTTGGACAGC-3’ |
| apchkf2 | Verify PAPa deletion | | 5’-ATCGGTATCATCAGTATCAGC-3’ |
| papbf1 | Deletion of PAPα allele | | 5’-TCGCTCGAGCCACTACTATTACTACTACC-3’ |
| papbr1 | Deletion of PAPα allele | | 5’-TCGCCCGGGCCTTACTATTAGAATAATC-3’ |
| papbf2 | Deletion of PAPα allele | | 5’-TCGCCCGGGATAGTTATGGAACATTGAGTG-3’ |
| papbr2 | Deletion of PAPα allele | | 5’-TCGCTCGAGTAAAATATATACAGCAAGTTGATAAC-3’ |
| mp5chk4 | Verify PAPα deletion | | 5’-TAATGTATATACATCTATATACC-3’ |
| pikaf1 | Deletion of PIKa allele | | 5’-TCGCTCGAGGAAATACCAGCCAGCATTCAAGAAAC-3’ |
| pikar1 | Deletion of PIKa allele | | 5’-TCGCCCGGGGGTTCAGTGTATTGATGATGTA-3’ |
| pikaf2 | Deletion of PIKa allele | | 5’-TTTGCGATAAGTTTGGCATCATATTC-3’ |
| pikar2 | Deletion of PIKa allele | | 5’-TCGCTCGAGTCCGATAAATGTAACTGTAATC-3’ |
| akchkf1 | Verify PIKa deletion | | 5’-TTGATCATACAAGCGAGTATAC-3’ |
| pikbf1 | Deletion of PIKα allele | | 5’-TCGCTCGAGGTTTCACCCTAAGTTCTTTC-3’ |
| pikbr1 | Deletion of PIKα allele | | 5’-TCGCCCGGGAGTTTATGATTACGTGGTCC-3’ |
| pikbf2 | Deletion of PIKα allele | | 5’-TCGCCCGGGCTAATGCAATGTCTATTCATTC-3’ |
| pikbr2 | Deletion of PIKα allele | | 5’-TCGCTCGAGTACCCTGTGTTATCATCTGGAAC-3’ |
| robpaf | RT-PCR | | 5’-CATCAAGGATTCGGAAACAC-3’ |
| robpaf2 | RT-PCR | | 5’-CCGTTGAATCCAGTATTAGGAGA-3’ |
| robpaf2 | RT-PCR | | 5’-CGTTCTCATGCTCCCCTAA-3’ |
| robpar | RT-PCR | | 5’-GTGGTTTCTTTTCGCCAAACC-3’ |
| robpαf2 | RT-PCR | | 5’-GACCCCTTAGATCGATTCATC-3’ |
| robpαr2 | RT-PCR | | 5’-TGTCCCATTTACCAAGAGTTAC-3’ |
| rpapaf2 | RT-PCR | | 5’-CAGCAACTAGATCTACCTATGC-3’ |
| rpapar2 | RT-PCR | | 5’-TATTGTTGTCATTGTTCTCC-3’ |
| rpapαf2 | RT-PCR | | 5’-AGCAGCATCAATTGATTCTGC-3’ |
| rpapαr2 | RT-PCR | | 5’-TTCCTCACCCCATCACTTGAC-3’ |
| rpikaf2 | RT-PCR | | 5’-ATAACGACAACATAGGTGTTC-3’ |
| rpikar2 | RT-PCR | | 5’-AAGAAACGCCTGTAGATACC-3’ |
| rpikαf2 | RT-PCR | | 5’-TCCTTCCCATATGAAGATTTGCA-3’ |
| rpikαr2 | RT-PCR | | 5’-GCACCGCATAGGACCAAAGC-3’ |
| TDH3F | RT-PCR | | 5’-CGAAGGTGCTCAAAAACACA-3’ |
| TDH3R | RT-PCR | | 5’-TCAACGGTCTTTTGGGTAGC-3’ |
| a1F | *MTL* genotyping & RT-PCR | | 5’-TTGAAGCGTGAGAGGCAGGAG-3’ |
| a1R | *MTL* genotyping & RT-PCR | | 5’-GATTAGGCTGTTTGTTCTTCTCG-3’ |
| alpha2F | *MTL* genotyping & RT-PCR | | 5’-CATGAATTCACATCTGGAGGCAC-3’ |
| alpha2R | *MTL* genotyping & RT-PCR | | 5’-AAGCAGCCAACTCAGGTGAC-3’ |
| pknmf1 | *Over & Misexpression* | | 5-TCAGTCGACATGCCAGTAGCACCCCATAA-3’ |
| pknmr1 | *Over & Misexpression* | | 5’-TCGGTCGACGTACTATAAATTCCTTGCGTTATC-3’ |
| papanmf1 | *Over & Misexpression* | | 5’-TCGGTCGACATGAACAATCAAGCATAC-3’ |
| papanmr1 | *Over & Misexpression* | | 5’-TCGGTCGACGCAGCTGCACTTACTGTTCCGA-3’ |
| cartpcrnew | *Testing genomic targeting* | | 5’-TACAATGTAGGCTGCTCTACACC-3’ |
| nmgpcr | *Testing genome targeting* | | 5’-GGACAAGCTCATTGAGTGAC-3’ |
